# Protocol for biomimetic tumoroid models by plastic compression using centrifugation

**DOI:** 10.1016/j.xpro.2025.103718

**Published:** 2025-03-26

**Authors:** Sam Devereaux, Ashley Lam, Anuja Upadhyay, Megan Fallows, Lianqi Qiu, Xueni Pan, Umber Cheema

**Affiliations:** 1 UCL Centre for 3D Models of Health and Disease, UCL Division of Surgery and Interventional Science, Faculty of Medical Sciences, Charles Bell House, University College London, 43-45 Foley Street, London W1W 7TY, UK; 2 Department of Computing, Goldsmiths, University of London, 25 St James’s, London SE14 6AD, UK

**Keywords:** Cell-based Assays, Cancer, Tissue Engineering

## Abstract

Here, we present a protocol for engineering biomimetic tumoroid models by plastic compression using centrifugation. We describe steps for generating multi-compartment tumor-stroma models by mixing cells into a collagen hydrogel crosslinked at 37°C and centrifuging the hydrogel. We then detail procedures for generating compartmentalized models and encapsulating the final layered hydrogel containing a 96-well tumor mass in a 24-well stroma. This protocol increases collagen density and improves mechanical properties of collagen hydrogels.

## Highlights

• Instructions for seeding cells, cultured in 2D, within a collagen I hydrogel
• Steps for centrifuging collagen hydrogels to remove fluid, producing dense collagen gels
• Guidance for embedding a tumor mass into a relevant stromal compartment
• Steps for centrifuging a complex collagen gel, making it ready for downstream applications

## Abstract (editor highlights)

Publisher’s note: Undertaking any experimental protocol requires adherence to local institutional guidelines for laboratory safety and ethics.

## Graphical abstract

**Figure:**
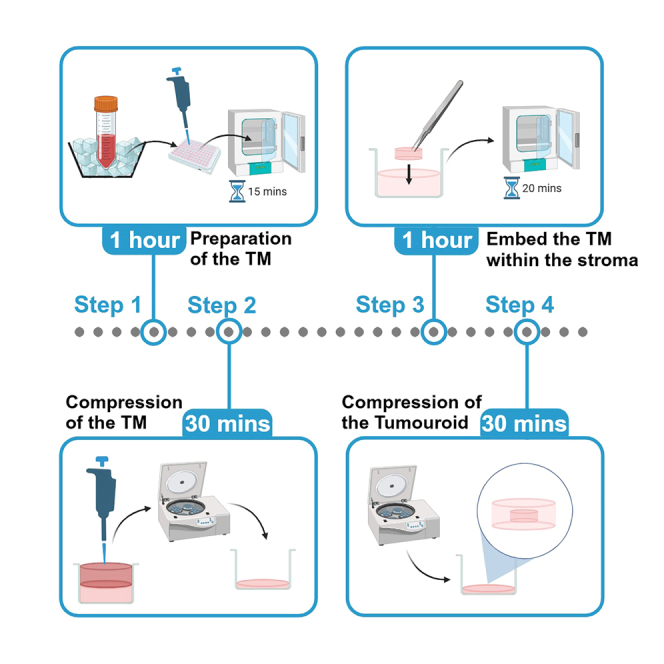

## Before you begin

Biomimetic, humanized 3D tissue models provide platforms for understanding disease mechanisms and for the testing of novel drugs and alternative therapeutic modalities. Recapitulation of the cellular, biophysical and biochemical facets of the tissue micro-environment is crucial. Collagen I is the predominant extracellular matrix (ECM) component of most tissues in the body. Collagen I hydrogels have been used extensively in research as a 3D biomimetic scaffold. A limitation of using standard collagen hydrogels is the low density and consequential mechanical properties. Hydrogels have a typical collagen concentration of 2–3 mg/mL, resulting in hydrogels which are 0.2–0.3% collagen and a high composition of liquid. Other than brain tissue, this is not biomimetic for most tissues in the body. Dense collagen scaffolds, formed by plastic compression (PC) of hydrogels have been used to engineer multiple tissue models including skin,^1^ nerve,^2^ muscle,^3^ and bone.^4^ It is possible to incorporate additional ECM proteins, including laminin and fibronectin into dense collagen gels.^5^^,^^6^

PC of cell-seeded collagen hydrogels was introduced in 2005 as a process for the controlled engineering of biomimetic scaffolds by the rapid removal of fluid from hyperhydrated hydro-gels.^7^ The PC method involves the physical compaction of cell-seeded collagen hydrogels sandwiched between support mesh layers and blotting elements for a period of 5 min, which resulted in the permanent loss of interstitial fluid, with a resultant increase in the collagen concentration of the gel to ∼6% collagen and cellular density. This patented technique was made available through the Real Architecture For Tissue (RAFT) system, which relied upon manipulation of the PC system through the application of absorbers directly onto collagen hydrogels, which increased in weight as liquid was absorbed from the underlying hydrogels. These methods rely upon blotting elements, are labor intensive and prone to human error during the fabrication process.

Herein, we describe a method to apply PC to cell-seeded collagen hydrogels by centrifugation. This process can be applied to multiple hydrogels set within standard multi-well plates (96, 24, 12, and 6-well format). A limitation of the absorption method^8^^,^^9^ for PC was the need to individually apply absorbers, resulting in variations between cultures. As the absorbers are single use, they impact the carbon footprint of the fabrication of tissue models. Our centrifugation method allows for simultaneous and reproducible PC of collagen hydrogels, resulting in dense scaffolds of consistent collagen percentage. This centrifugation process does not impact cell viability significantly, results in increased collagen and cellular density, and renders greater biomimicry to human tissue.

Furthermore, we detail a procedure to engineer multi-compartment tumor-stroma models, by generating a tumor mass (TM) through PC that can be embedded in more complex stromal compartments. This provides spatial segregation of cells to mimic the native tumor-stroma, and allows for the study of disease progression, personalized medicine, and the interaction of relevant stromal cells. These biomimetic 3D *in vitro* models are referred to as “tumoroids.” The mechanical properties of the tumoroids are described along with the viability and growth of 2 breast cancer cell lines as an example. This protocol can be adapted to include various cell types inside the TM and stromal compartments to model different tumors.

This protocol describes embedding tumor and other cell types into collagen scaffolds. Cells should be cultured in cell-dependent media. The same media should be used for subsequent 3D culture. If multiple cell lines are embedded that require different growth mediums, it is recommended to mix the mediums corresponding to cell number ratios. Please refer to the reagent volume guide for the exact volumes needed. This method has also been used to develop models for a variety of cell types shown in Table 1.

**Table 1:** Current cancer types and cell lines that have been investigated in the tumoroid model

Cancer type	Cell line
Breast	MDA-MB-231
Breast	MCF-7
PDAC	PANC0203
PDAC	ASPC1
Renal	ACHN
Renal	Caki-2
Renal	786-O

## Key resources table

**Table:** 

REAGENT or RESOURCE	SOURCE	IDENTIFIER
**Chemicals, peptides, and recombinant proteins**		

Sodium hydroxide	Honeywell/Fluka	Cat# 011-002-00-6
MEM (10x)	Gibco	Cat# 11570566
Collagen (rat tail) type I	First Link	Cat# 60-30-810
HEPES buffer (1 M)	Gibco	Cat# 15630-056
RPMI 1640 + L-glutamine	Gibco	Cat# 21875-034
Fetal bovine serum (FBS)	Gibco	Cat# 11550356
Penicillin-streptomycin solution	Gibco	Cat# 11548876
PBS	Gibco	Cat# 10010023
Trypsin-EDTA	Gibco	Cat# 25200056
Trypan blue	Invitrogen	Cat# 10702404

**Critical commercial assays**		

PrestoBlue cell viability reagent	Invitrogen	Cat# A13261

**Deposited data**		

Raw and analyzed	This paper	N/A

**Experimental models: Cell lines**		

MCF-7	ECACC	Cat# 86012803
MDA-MB-231	ECACC	Cat# 92020424

**Software and algorithms**		

ImageJ	Open-source	https://imagej.net/ij/download.html
GraphPad Prism 10	Dotmatics	https://www.graphpad.com/updates

**Other**		

Centrifuge 5910 R	Eppendorf	Cat# 16622842
FreeZone 2.5 L −50°C benchtop freeze dryer	Labconco	Cat# 700202050
Kinexus Prime pro+ rheometer	Netzsch	N/A
Zeiss Axio Observer	Zeiss	Apotome.2
Tecan plate reader	Tecan	Infinite M Plex
50 mL Falcon tubes	Corning	Cat# 430829
96-well culture plates	Corning	Cat# 3599
24-well culture plates	Corning	Cat# 3524
Surgical forceps	DBIO GmbH	Cat# DBF1001

## Materials and equipment

**Table:** Neutralizing solution

Reagent	Final concentration	Amount
Sodium Hydroxide (10 M)	16.5%	0.825 mL
HEPES (1 M)	83.5%	4.165 mL
**Total**	**100%**	**4.99 mL**

Storage conditions: Store at 4°C for up to 11 days.

**CRITICAL:** Sodium Hydroxide is a danger, may be corrosive to metals and causes severe skin burns and eye damage. Take precautions by wearing protective gloves, clothing, eye protection, and face protection.

**Table:** RPMI cell culture medium

Reagent	Final concentration	Amount
RPMI 1640 + L-Glutamine	89%	445 mL
FBS	10%	50 mL
Penicillin-Streptomycin	1%	5 mL
**Total**	**100%**	**500 mL**

Storage conditions: Store at 4°C for up to 6 weeks.

**Table:** Collagen mixture

Reagent	Final concentration	Amount
Collagen (2 mg/mL)	80%	N/A
MEM (10x)	10%	N/A
Neutralizing Solution	5.83%	N/A
Culture media containing cells	4.17%	N/A
**Total**	**100%**	**N/A**

Storage conditions: Make fresh and use immediately.

**Table:** Reagent volume guide for making tumoroids in 96-well plates

No. of gels	10x MEM (mL)	Collagen (mL)	Neutralizing solution (mL)	Cells in media (mL)	Total volume (mL)
1	0.12	0.96	0.070	0.050	1.2
2	0.15	1.2	0.087	0.063	1.5
3	0.17	1.4	0.099	0.071	1.7
4	0.20	1.6	0.116	0.084	2.0
5	0.22	1.8	0.128	0.092	2.2
6	0.24	1.9	0.139	0.101	2.4
7	0.27	2.2	0.157	0.113	2.7
8	0.29	2.3	0.168	0.122	2.9
9	0.32	2.6	0.186	0.134	3.2
10	0.34	2.7	0.197	0.143	3.4

**Table:** Reagent volume guide for making tumoroids in 24-well plates

No. of gels	10x MEM (mL)	Collagen (mL)	Neutralizing solution (mL)	Cells in media (mL)	Total volume (mL)
1	0.33	2.6	0.191	0.139	3.3
2	0.46	3.7	0.267	0.193	4.6
3	0.59	4.7	0.342	0.248	5.9
4	0.72	5.8	0.418	0.302	7.2
5	0.85	6.8	0.493	0.357	8.5
6	0.98	7.8	0.568	0.412	9.8
7	1.1	8.8	0.638	0.462	11
8	1.2	9.6	0.696	0.504	12
9	1.5	12	0.870	0.630	15
10	1.6	12.8	0.928	0.672	16

All image quantification was carried out using Image J analysis software. Other analysis and graph production was performed on GraphPad Prism 10. All statistical details can be found in the relevant figure legends.

## Step-by-step method details

### Preparation of the tumor mass

**Timing: 1.5 h**

This section describes the process of setting tumor cells within a solid Collagen I gel and its subsequent compression using centrifugation. All steps detailed in this protocol should be performed in a biosafety cabinet to ensure sterility.

1. On ice, mix collagen I and MEM, swirl gently by hand to avoid bubbles, do not vortex. View the reagent volume table for exact quantities depending on the number of tumoroids being made.

***Note:*** Mixing by pipette will produce unwanted bubbles that will interfere with downstream imaging.

**Figure 1:**
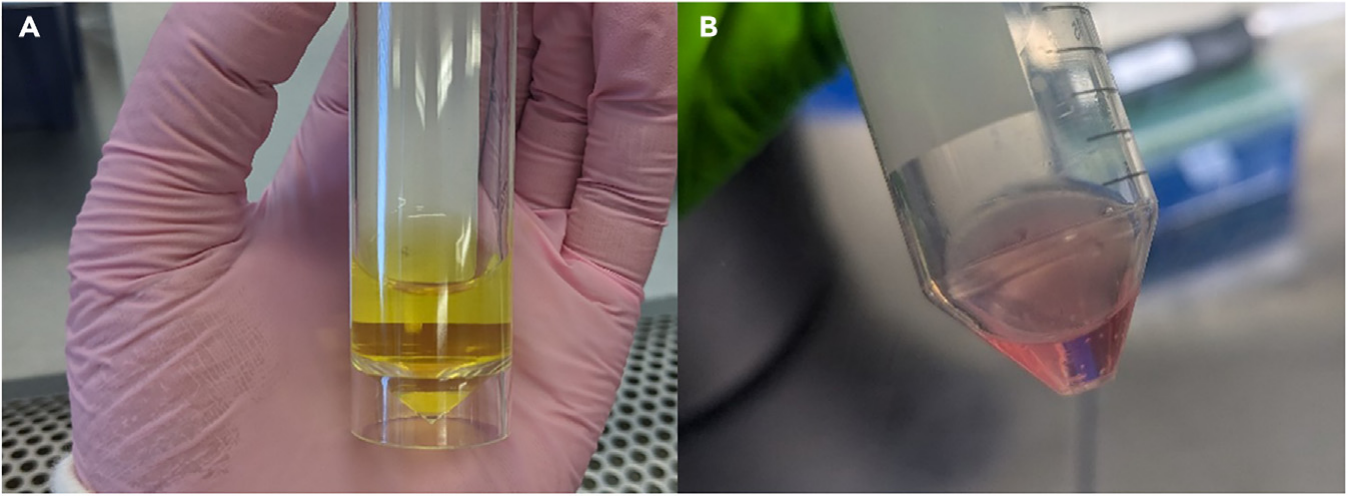
Expected color change during neutralization of Collagen mixture The approximate color change expected following the dropwise addition of the neutralizing solution. The color should change from orange (A) to salmon pink (B).

2. Add neutralizing solution drop by drop and mix by swirling gently. The mixture should turn a salmon pink color. See Figure 1B for a color guide.

**Figure 2:**
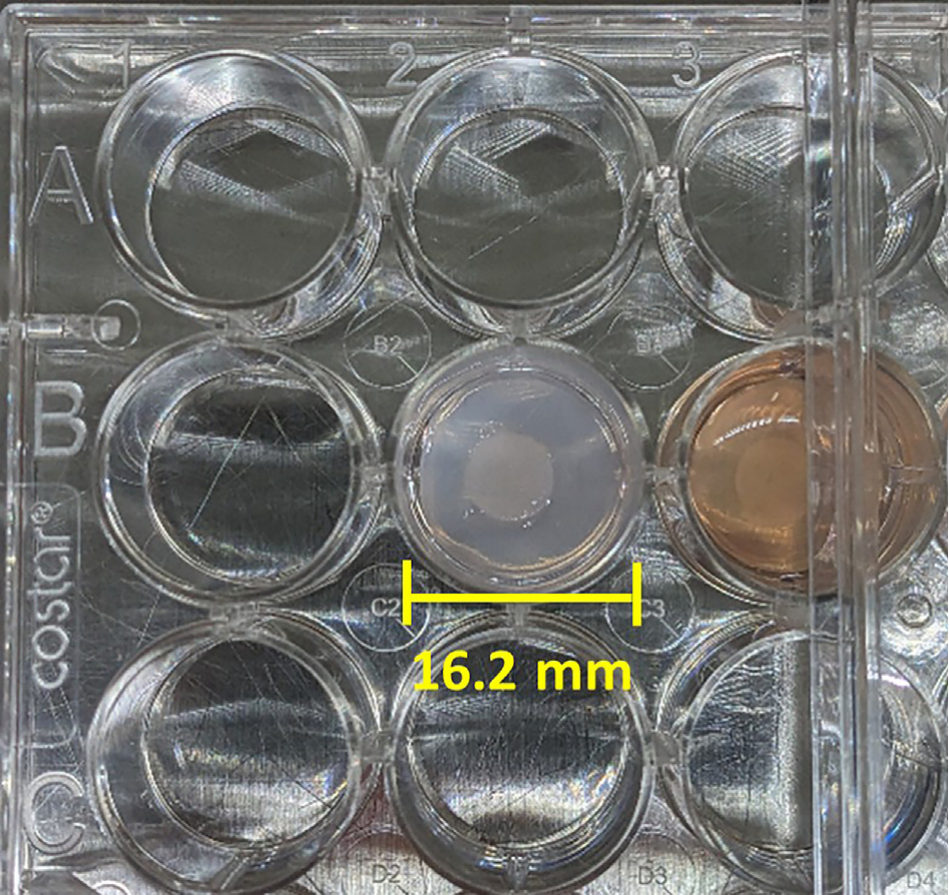
A compressed tumoroid with TM and surrounding stromal compartment, following the centrifugation process

***Note:*** Neutralizing solution could have batch-to-batch differences it is therefore advised to add the neutralizing solution slowly, if the mixture turns a hot pink color, you have added too much neutralizing solution and should remake the gel mix as it may be cytotoxic and may hinder the crosslinking of the gel if not appropriately pH balanced. The target pH should be around 7.2–7.4.

3. Leave the mixtures on ice with the lids off for approximately 20 min to allow bubbles to exit.
4. Add media containing 5 x $10^{4}$ of the cell type of interest and mix well (avoid creating bubbles).

***Note:*** 5 x $10^{4}$ cells is the recommended starting quantity per TM. This means the overall cell number added to the mixture should correlate to x∗(5 x $\;10^{4})$, where x is the number of gels being created. It is important for users to validate cell attachment and viability using various cell viability measurements (such as PrestoBlue). Cell number should be optimized according to cell-type and down-stream applications.

5. Pipette 240 μL of the gel mixture into each well of a 96-well plate (per TM being made). Ensure gels are pipetted into central columns of the plate (do not pipette gels into the border wells of the plate) and work in parallel with 2 plates for centrifugation or create a balance.

***Note:*** It was found that pipetting gels into wells around the periphery of the plate resulted in less uniform compression. Therefore, it is suggested to position the gels as centrally to the plate as possible.

6. Incubate for 15 min at 37°C.
7. Add 60 μL of media on top of the solidified gel.
8. Using sterile tweezers, dislodge the gel by carefully scraping the tweezers around the circumference of the well. The gel should become free-floating in the well. Be careful to avoid damaging the gel.
9. Spin the plates containing the gels in a centrifuge at 4347 RCF for 15 min, ensuring the centrifuge is balanced. During this step the gels will undergo plastic compression.

***Note:*** Optimization was carried out using an Eppendorf 5910 R Centrifuge and rotor. For this centrifuge, 4347 RCF corresponded to 4500 RPM.

10. Remove the excess liquid from on top of the compressed gels with a pipette, leaving behind only the thin compressed TM.

### Preparation of the stroma and insertion of the TM

**Timing: 1.5 h**

This section describes creating the stromal compartment and carefully embedding the previously made TM using tweezers, followed by compression by centrifugation to reach the completed tumoroid (Figure 2).

11. Make the gel mixture in the same fashion as for the TM but for 24-well plate size (refer to the reagent volume guide). This mixture may be acellular or contain other cells of interest (e.g., fibroblasts).

***Note:*** The gel mixture can be made at the same time as the 96-well size gels. If done this way, ensure the mixture is kept on ice until use to prevent collagen cross linking.

12. Add half the single gel volume (650 μL) into each well of a 24-well plate. Ensure gels are pipetted into central columns (leaving the border wells of the plate empty) of the plates and work in parallel with 2 plates for centrifugation or create a balance.
13. Incubate for 20 min at 37°C.
14. Using sterile tweezers, add a compressed TM on top of each solidified gel mixture, then add the remaining volume for a single gel (650 μL) to each well.

***Note:*** When pipetting the gel mixture on top of the TM, be slow and careful to avoid moving the TM off-center.

15. Incubate for 20 min at 37°C.
16. Add 1.3 mL of media (relevant to the cell type chosen) on top of the compartmented gel and use sterile tweezers around the edge of the well to lift the gel so it is free-floating.
17. Spin at 4347 RCF for 15 min.
18. Remove excess liquid leaving only the compressed tumoroid.
19. Add 1 mL of media on top of each tumoroid.
20. Place in a cell culture incubator and replace 50% of the spent media with fresh media every 48 h.
21. Perform any downstream experiments relevant to the specific project aims. For example, add anti-tumor drugs and assess their efficacy by immunofluorescence imaging or flow cytometry, etc.

## Expected outcomes

The embedding of different cell types, with spatial segregation, to model tumors with varying degrees of complexity can produce numerous avenues for exploring cancer progression and testing drug efficacy in a more robust environment than 2D monolayers. This protocol can be scaled to produce large amounts of simple or complex tumoroids in either 96-, 24-, or 6-well plates with one or more spatial compartments, depending on the desired output.

### Quantification of biomechanical properties

The collagen percentage of these models can be quantified through dry/wet weight analysis. Dry weight can be obtained following freeze drying for 24 h and collagen density can be calculated using the formula: $\frac{Dry\;Weight\;\left(g\right)}{Wet\;Weight\;\left(g\right)}\;*100$.

**Figure 3:**
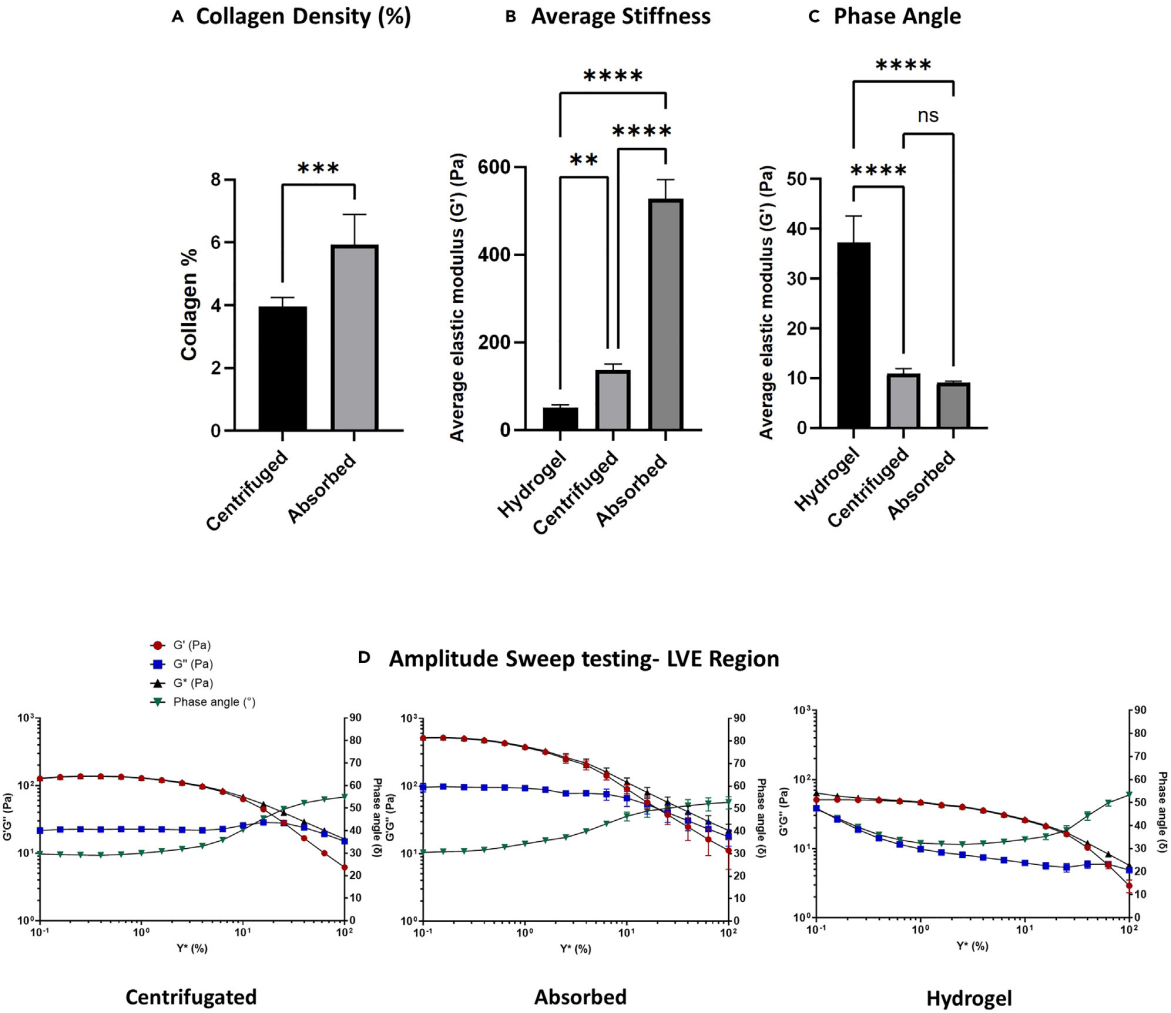
Biomechanical profiles of collagen hydrogel, absorbed gels and centrifuged gels (A) Quantification of collagen density in absorbed (*n* = 5, sd = 0.9485) and centrifuged (*n* = 8, sd = 0.2966) gels as percentages (w/v), *p* = 0.0095. (B) Average shear elastic modulus of different gels: Hydrogel, *n* = 3. Centrifuged, *n* = 3. Absorbed, *n* = 3. For ∗∗*p* = 0.0068. For ∗∗∗∗*p* < 0.0001. Error bars represent SEM. (C) Phase angle graph of gels, for significant data, for ∗∗∗∗*p* < 0.0001. (D) Amplitude sweep graphs of different gels with end of LVE region annotated with a red dotted line. All tests were conducted using a roughened immersion well geometry and a 20 mm roughened steel plate top geometry.

Tumoroids’ compressed with centrifugation should produce a collagen density of ∼4% as shown in Figure 3. This is slightly lower than the ∼6% offered by the original mechanical load absorption.^10^

A beneficial property of this model is offering a mechanical “stiffness” that is relevant to certain human tissues. Tissue stiffness and its viscoelastic behavior are crucial properties of biological tissues. This is determined by the structural properties and composition of the ECM. Biomechanical homeostasis is essential for maintaining function and resilience in various organs. Viscoelasticity is a property of materials which exhibit viscous (fluid like) and elastic (solid like) behavior, allowing tissues to absorb and dissipate energy to help with structural integrity and movement.

Stiffness is defined as a material’s ability to resist deformation and measured as the elastic modulus (Pa). This property is expressed as the amount of stress (compressive, Ce or shear) divided by the strain that is induced by the stress. Elasticity can be quantified using various methodologies such as shear rheology, which assesses the deformation and flow behavior of materials to determine mechanical properties. Amplitude sweep testing determines the shear elastic modulus (Pa), the linear viscoelasticity (LVE) region and the phase angle as a calculated value between 0 and 90 that reflects a material’s inherent structure. This test measures a material’s response to increasing strain rates from 0-100% to get a bulk profile of its behavior. The linear viscoelasticity region denotes the range in which the stress is equal to the induced strain and deformation is reversible, the end of the LVE region highlights the maximum stress needed to trigger material failure with irreversible deformation.

Shear elasticity is measured using the equations

$$\tau \;\left(shear\;stress\right)=F\;\left(shear\;force\;in\;N\right)/\;A\;\left(shear\;area\;in\;m^{2}\right)$$

G(shearmodulus)=τ(shearstress)/γ(shearstrain)

Oscillatory shear testing measures G∗ (complex shear modulus), an overall value for the shear elastic modulus (G′) and shear viscous modulus (G″), and phase angle (δ) using the equations below:

$$G^{\prime }=G^{*}\times \cos\;\;\cos\;\delta$$

$$G^{\prime \prime }=G^{*}\times \sin\;\;\sin\;\delta$$

$$\tan\;\;\tan\;\delta =G^{\prime \prime }/G^{\prime }$$

The PC method using absorption generates collagen gels with an average elastic modulus of 527.73 Pa ± 35.04 (1 s.d.) (Figure 3B) and a collagen density of 6% (Figure 3A). In comparison, the uncompressed hydrogels measure 51.26 Pa ± 5.06 (1 s.d.) and 0.2% collagen density. Centrifugation increases the stiffness to 136.70 Pa ± 11.66 (1 s.d.) and measures at 4% collagen density. While mechanical load compression fabricates stiffer gels, centrifugation also provides a stable and elastic solid like environment that holds more biological relevance than uncompressed hydrogel models. This is further supported by the phase angles of compressed and centrifuged gels measuring at 10.83 ± 1.03 and 9.37 ± 0.21, respectively. This low phase angle indicates more elastic solid energy in the material whereas the hydrogels hold more viscous potential, with an average phase angle of 27.58 ± 2.07 (Figure 3C). The LVE region (red line) is longer in the hydrogels as the viscous energy it holds enables it to withstand higher strain rates before reaching irreversible deformation, in comparison to the absorbed and centrifuged models (Figure 3D).

### Tumor growth and invasion

**Figure 4:**
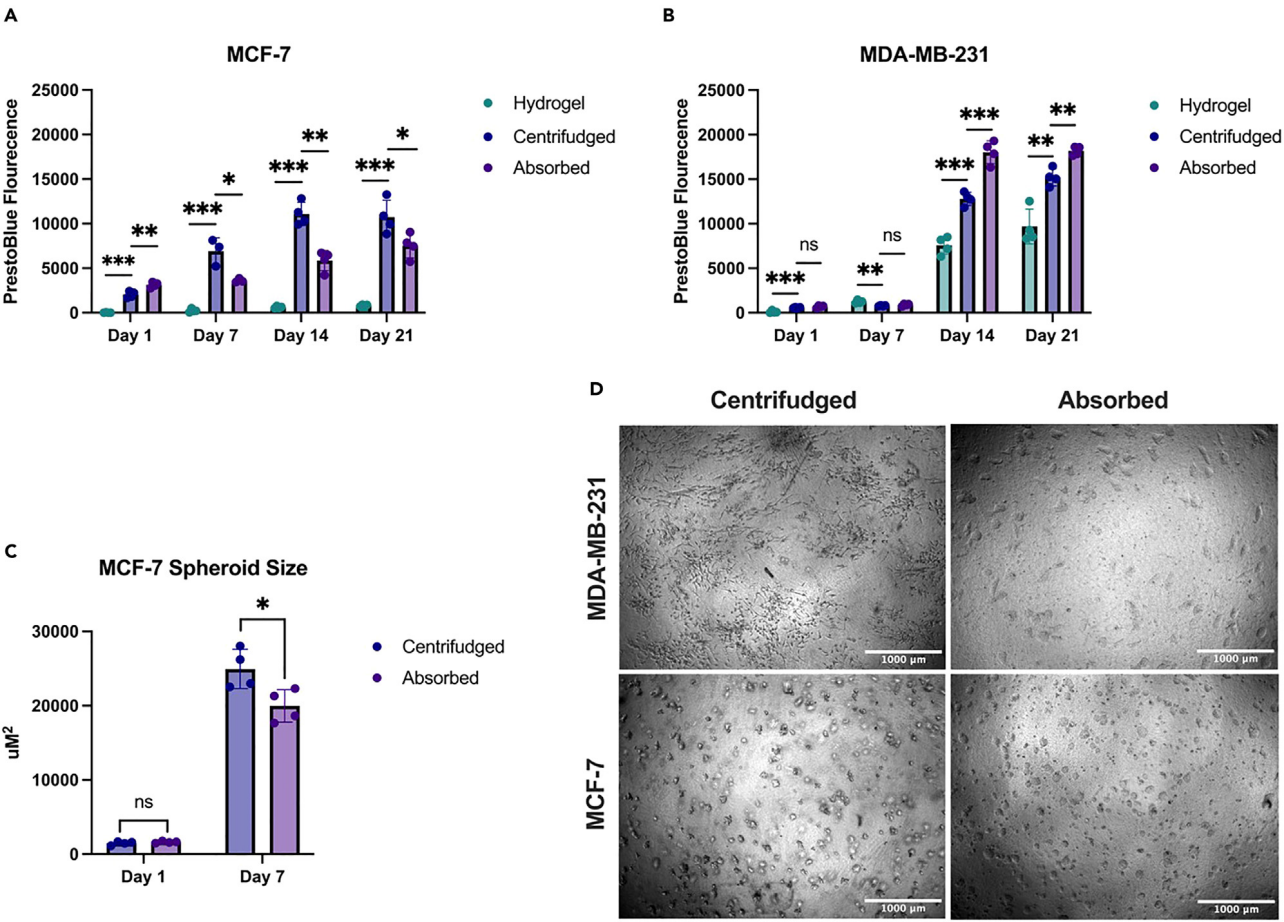
Cancer cell growth within tumoroids (A–C) Prestoblue fluorescence of centrifuged and absorbed tumoroids measured every 7 days for 21 days of MCF7 (A), *n* = 4, and MDA-MB-231 cells (B), *n* = 4. Spheroid size of MCF-7 cells (C) centrifuged and absorbed tumoroids at day 1 and day 7 (*n* = 4) quantified in Image J from phase contrast images. (D) Representative phase contrast images of morphology of MDA-MB-231, and MCF-7 cells in absorbed and centrifuged tumoroids respectively (Scale bar represents 1,000 μm). ∗*p* < 0.05, ∗∗*p* <0.01, ∗∗∗*p* < 0.001.

Cancer cells will proliferate and form spheroids at a comparable rate whether they are compressed by mechanical load absorption or centrifugation as shown in Figure 4. Cell viability over the course of the 21-day culture was determined by Prestoblue (Thermo Fisher Scientific, A13262) measurements every 7 days. Prestoblue results (Figures 4A and 4B) demonstrated sustained cell viability and proliferation over the entire culture period. The compressed matrices (centrifuged and absorbed) provide an environment which enhances the proliferation of both the mesenchymal (MDA-MB-231) and the epithelial (MCF-7) cell line. The centrifugation method was demonstrated to have a significantly higher cell viability measurement than its hydrogel and absorbed counterparts’ day 14 onwards in both cell lines (Figure 4A and 4B).

In addition, we quantified spheroid size in MCF-7 cells. Cancer cells form spheroids naturally when grown in a 3D context and this self-organization into a spheroid morphology mimics the way tumors grow in living tissues and can influence tumor biology, metastasis and drug resistance. MCF-7 cells formed slightly larger spheroids in the centrifuged model than absorbed model at day 7 (Figure 4C). In both the absorbed and centrifuged tumoroid models of MCF-7 (epithelial) and MDA-MB-231 (mesenchymal) cell lines showed comparable morphology (Figure 4D).

We have detailed the steps to fabricate our model, where the cancer compartment (TM) is encapsulated within a larger stromal compartment. By spatially segregating these two compartments, the model is engineered to mimic the organization of *in vivo* solid tumors. This structure facilitates the formation of nutrient, chemical, and oxygen gradients, enabling cancer-stromal interactions in a physiologically relevant manner.^11^^,^^12^^,^^13^ Additionally, this arrangement allows for the measurement of cancer invasion and migration from the TM into the stromal compartment, providing a functional assay to evaluate cancer behavior and biology.

**Figure 5:**
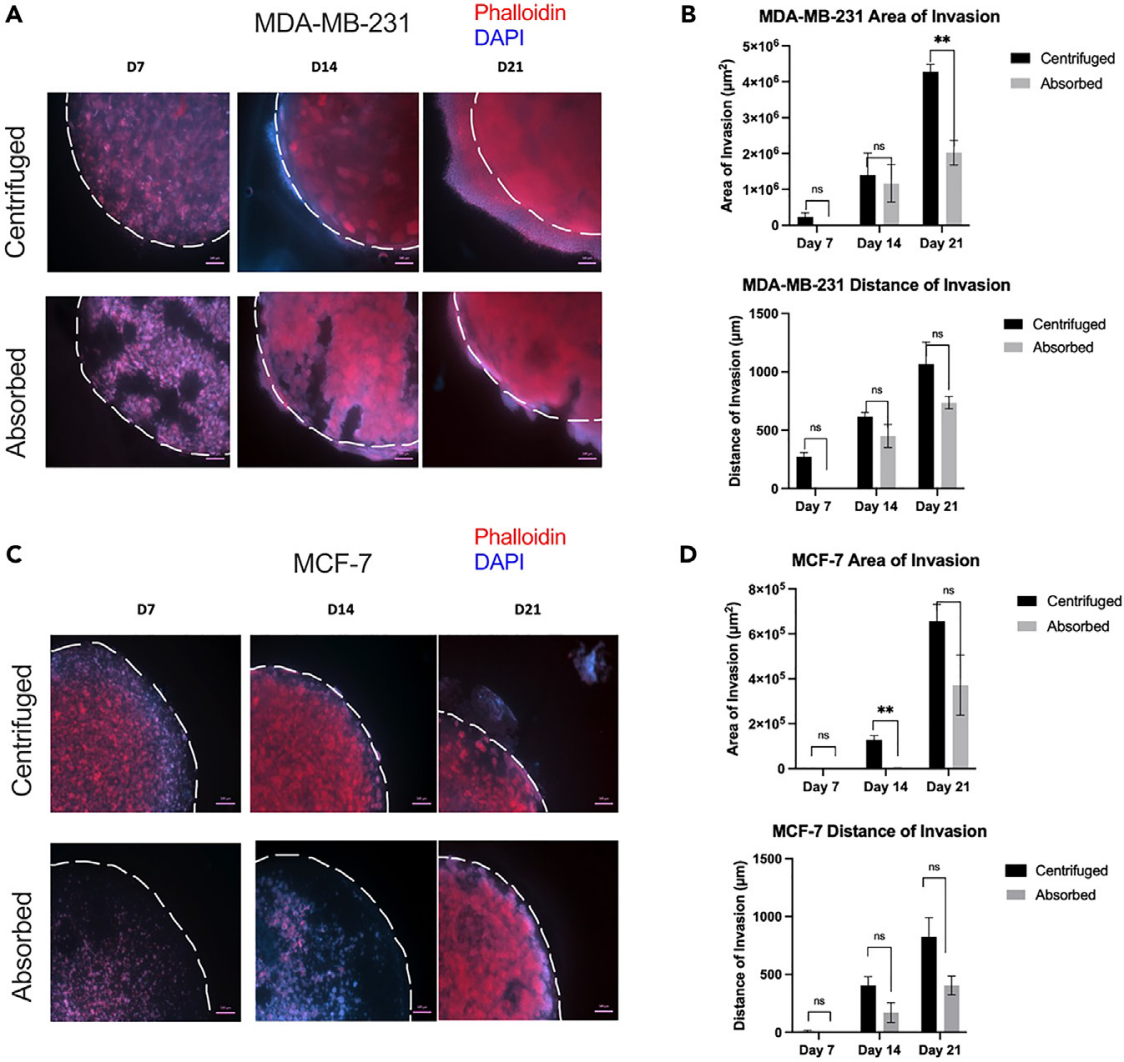
Cancer invasion in tumoroid models (A) Representative images (*n* = 3) at days 7, 14 and 21 of centrifuged and absorbed tumoroids with 50K MDA-MB-231 cells seeded in the TM embedded in an acellular stroma. All images in this figure were fixed, blocked, stained with Phalloidin (red) and DAPI (blue), imaged on an Axio Observer Zeiss microscope, and quantified using ImageJ. Scale bar represents 500 μm. (B) Quantifications of MDA-MB-231 invasion distance and surface area into the stromal compartment. Invasion markers were confirmed by qPCR (data not shown). (C) Representative images (*n* = 3) at days 7, 14 and 21 of centrifuged and absorbed tumoroids with 50K MCF-7 cells seeded in the TM embedded in an acellular stroma. (D) Quantifications of MCF-7 invasion distance and surface area into the stromal compartment. ∗∗*p* < 0.01.

We present the invasion and migration patterns of mesenchymal (MDA-MB-231) and epithelial (MCF-7) breast cancer cell lines (Figure 5). Both cell lines exhibited continuous proliferation and invasion throughout the 21-day culture period. The invasion distance and surface area of MDA-MB-231 and MCF-7 cells were comparable between the absorbed and centrifuged tumoroid models (Figures 5A–5D), though at certain time points the centrifugation methods showed more invasion than the absorption method. The invasion distance and surface area of MDA-MB-231 cells (in both absorbed and centrifuged methods) were consistently greater than those of MCF-7 cells, aligning with literature that MDA-MB-231 is a more invasive mesenchymal cell line.

The centrifugation method to generate dense collagen tumoroid models, much like its predecessor—the RAFT absorption PC method—offers a well-defined tumor-stromal border, allowing for the quantification of cancer invasion and cell motility. This model allows for the easy extraction of DNA, RNA, and protein, enabling analyses such as sequencing, RT-qPCR, and Western blotting. Additionally, it can be used for live-cell imaging or fixed for immunofluorescence imaging and analysis. This protocol described herein is a more accessible and sustainable method of generating tumoroids compared to the previous RAFT system and allows a higher throughput. A wide range of cell lines and cell types have been utilized in this model, demonstrating its versatility and significant potential for modeling various cancer types and their microenvironments.

## Limitations

The centrifugation method does result in a reduced collagen density and stiffness compared to the previous RAFT absorber system, although both are still significantly higher than hydrogel models offering improved biological relevance. However, phase angle demonstrates that these centrifuged models show similar properties as an elastic solid material to RAFT models, unlike hydrogels which have more viscous potential. There is potential for some cells to be affected by the high centrifugal force, however so far this has not been observed, indicating the 3D structure offers protection compared with centrifuging 2D monolayers.

## Troubleshooting

### Problem 1

Not achieving the correct color change when adding neutralizing solution in step 2.

### Potential solution

• Ensure the neutralizing solution has been freshly made within the last 11 days, as it loses potency over time.
• Add the solution in a dropwise fashion. It is acceptable to add slightly less or more to achieve the correct color.
• If too much is added and the mixture turns bright pink, restart the process.

### Problem 2

Creating bubbles while mixing the reagents in steps 1, 4 or 11.

### Potential solution

• Do not mix the reagents by pipette or vortex.
• It is recommended to swirl the reagents in a falcon tube by hand in a rotational manner, do not shake or turn the tube upside down.
• Large bubbles on the surface can be removed by pipette and the mixture can be left with the lid left off to aid release of small bubbles within the mixture.

### Problem 3

Non-uniform plastic compression producing uneven tumoroids in steps 5 or 12.

### Potential solution

• When pipetting the mixture into a 96 or 24 well plate, ensure they are placed as centrally to plate as possible. Avoid putting the tumoroids in the wells around the edges of the plates. For large scale productions, spread them across multiple plates if necessary to achieve this.

### Problem 4

Difficulty placing the TM into the stromal compartment in step 14.

### Potential solution

• Use sterilized forceps for this process. When removing the TM from the 96 well plate, folding can occur which can be difficult to undo. It is recommended to place the TM in a pool of media in a larger container, for example in an upturned lid of the 96 well plate. In most instances this is enough for the TM to unfold. Otherwise, use two forceps to flatten out the TM and then transfer it to the stromal compartment.
• When putting the TM into the stroma, flatten it over the surface and then add the remaining mixture very gently to avoid displacing the TM.

### Problem 5

Cells not growing as expected or dying early.

### Potential solution

• Seeding densities in this protocol are only suggestions and should be optimized depending on chosen cell types. For instance, if cells are slow growing in 2D monoculture or require cell-to-cell contact, then higher seeding densities should be considered.
• If using multiple cell types in the same model, it is recommended to test the viability of each individually in 3D before combining.
• If using multiple cell types in the same model, optimization of the combined media may be required in 2D first.

## Resource availability

### Lead contact

Further information and requests for resources and reagents should be directed to and will be fulfilled by the lead contact, Umber Cheema (u.cheema@ucl.ac.uk).

### Technical contact

Technical questions on executing this protocol should be directed to and will be answered by the technical contact, Umber Cheema (u.cheema@ucl.ac.uk).

### Materials availability

This study did not generate new unique reagents.

### Data and code availability

This study did not generate any datasets/code.

## Acknowledgments

The authors would like to give thanks to their funding bodies: the UKRI MRC (MR/W006774/1) (S.D.) and UCB Pharma (A.U.). The graphical abstract was created in BioRender (Devereaux, 2025, https://BioRender.com/t65l774).

## Author contributions

U.C. and S.D., protocol conceptualization. S.D., A.U., and A.L., method testing and development. A.U., rheology and phase angle experiments and analysis. M.F. and A.L., tumor growth and invasion imaging and analysis. S.D., U.C., A.L., and A.U., manuscript writing. L.Q. and X.P., cover art. U.C., scientific work supervision.

## Declaration of interests

The authors declare no competing interests.
